# Supplementary material for: Investigating immune and non-immune cell interactions in head and neck tumors by single-cell RNA sequencing
Source: Nat Commun. 2021 Dec 17;12:7338. doi: 10.1038/s41467-021-27619-4 (PMC8683505; doi:10.1038/s41467-021-27619-4)
Supplement: Supplementary file 1 — Supplementary Information [file 41467_2021_27619_MOESM1_ESM.pdf]

Supplementary Figure 1

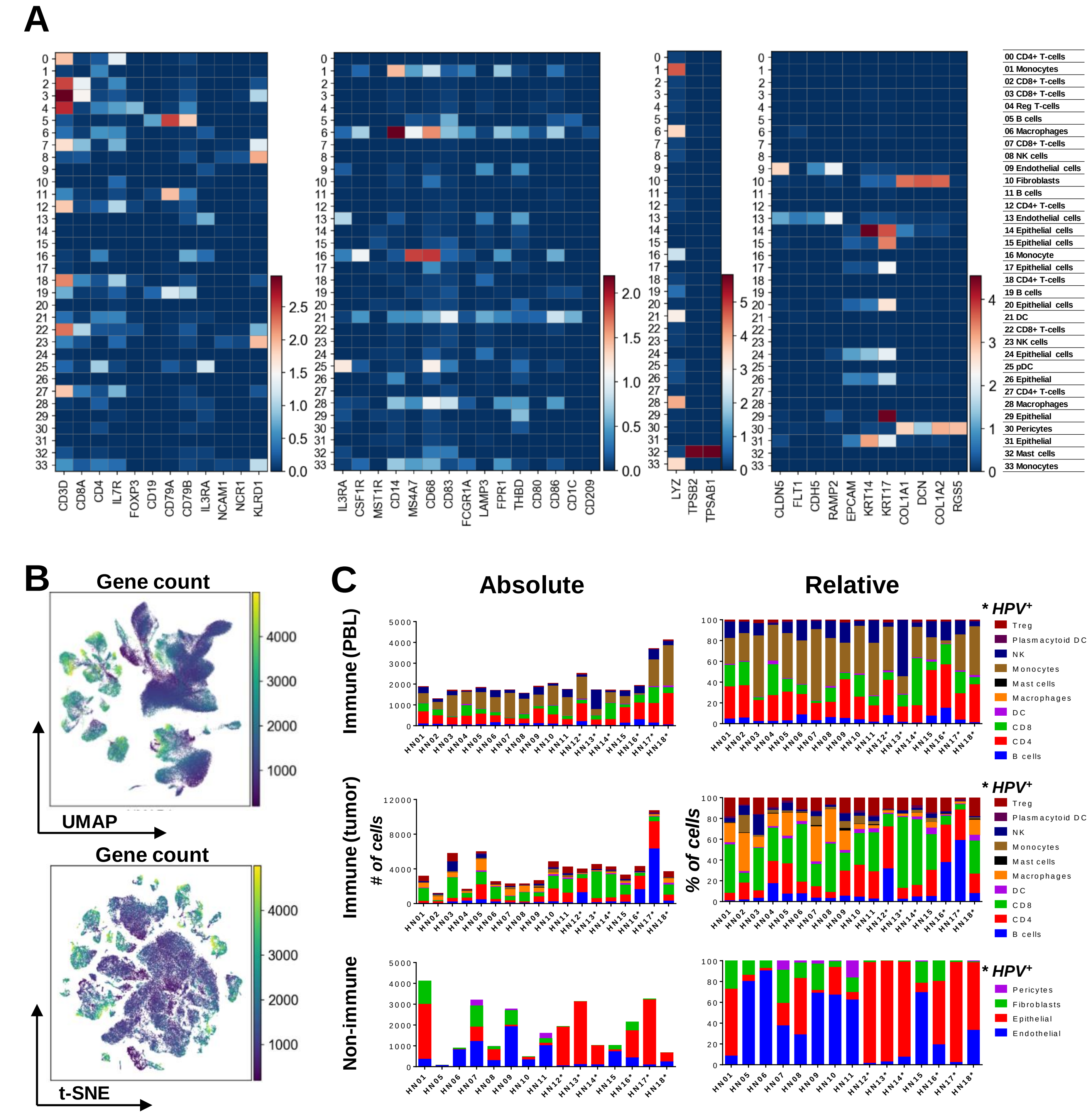

Supplementary Figure 1: Extended data on cell type allocation and distribution. (A) Matrix plot showing expression of marker genes used for cell type assignments depicted in **Fig. 1C**. Color bars depict average gene expression. (B) UMAP and t-SNE plots showing the number of genes per cell. (C) Absolute counts and relative proportions of each cell type found in circulation (PBL) and tumors (PTPRC/CD45+ immune and PTPRC/CD45- non-immune cells).

Supplementary Figure 2

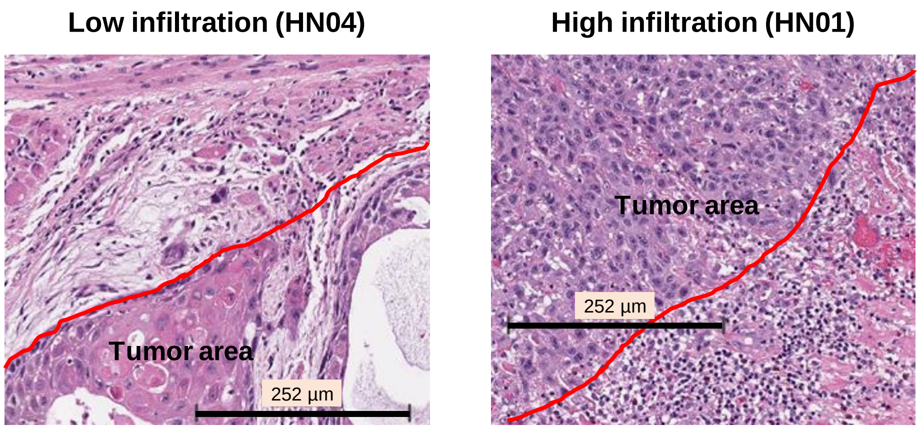

Supplementary Figure 2: Extended data on inflammation scoring. Representative H&E images (n=17) for tumors with low (HN04) and high (HN01) infiltration scores highlighting the differences between the leukocyte infiltrates.

Supplementary Figure 3

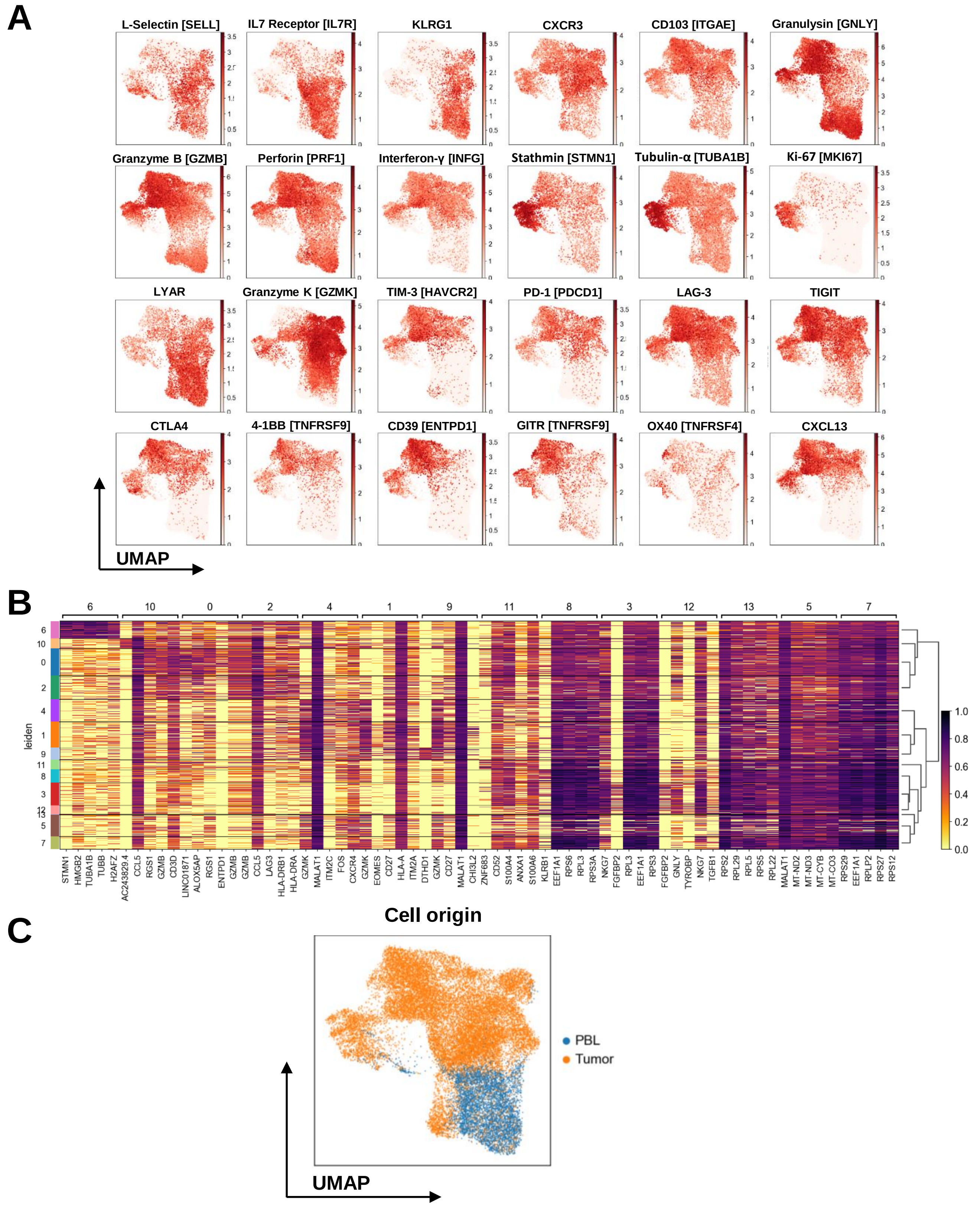

Supplementary Figure 3: Extended data on CD8⁺ T-cell substates. (A) UMAP plots showing genes associated with CD8⁺ T-cell differentiation, activation and proliferation (n=18 patients). Colored scale bars indicate normalized gene expression (B) Heatmap showing top 5 genes characterizing each CD8⁺ T-cell cluster. Color scale depicts scaled gene expression levels. (C) UMAP showing the sample of origin for CD8⁺ T-cells (PBL vs. tumor)

Supplementary Figure 4

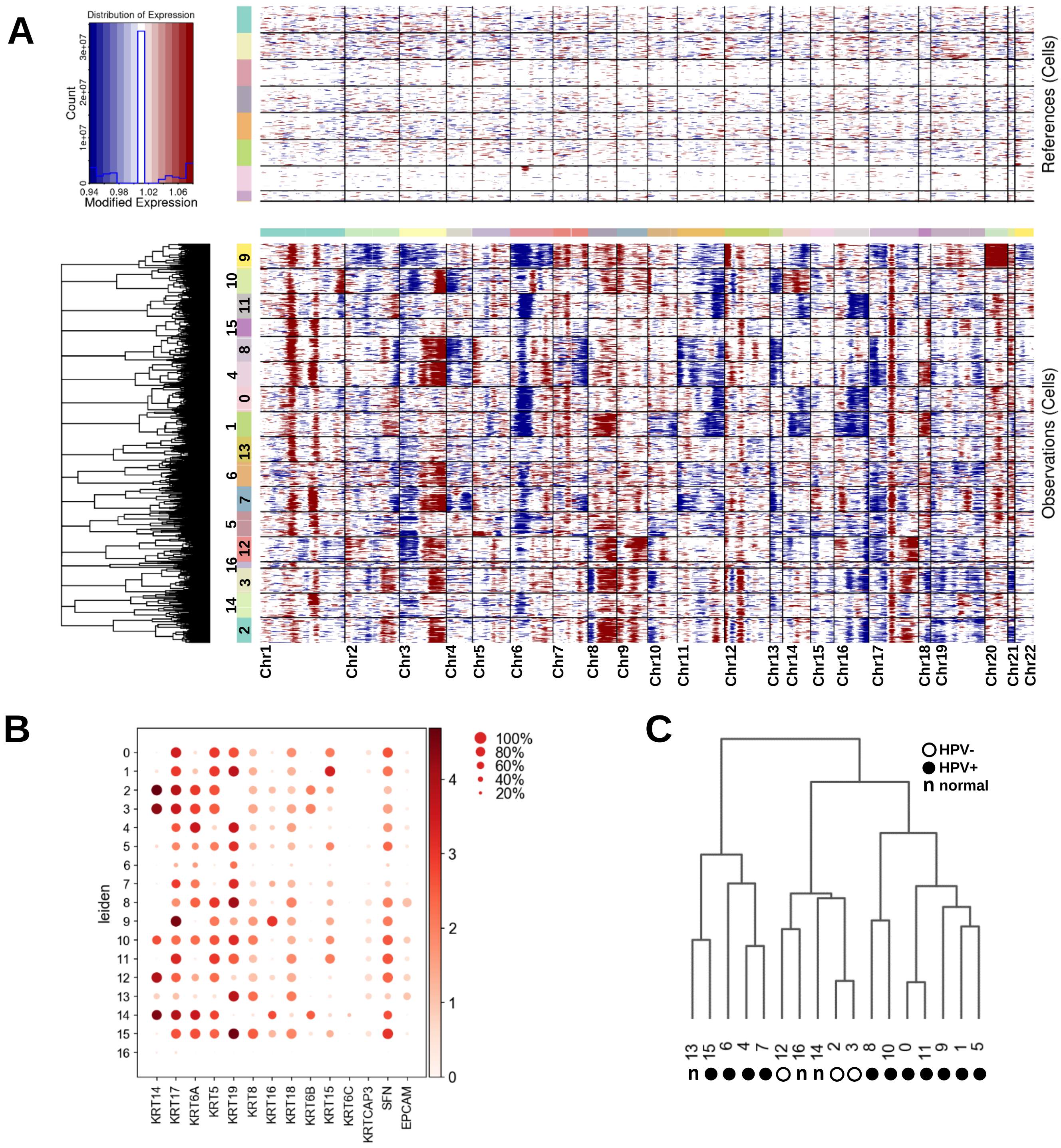

Supplementary Figure 4: Extended data on epithelial cell characterization (n=15 patients) (A): Comparison between PBL and epithelial cells to establish malignancy using InferCNV. (B) Dot-plot showing keratin and related marker expression levels in epithelial cell clusters. Color bar indicates normalized gene expression (C) Cluster hierarchy of epithelial clusters.

Supplementary Figure 5

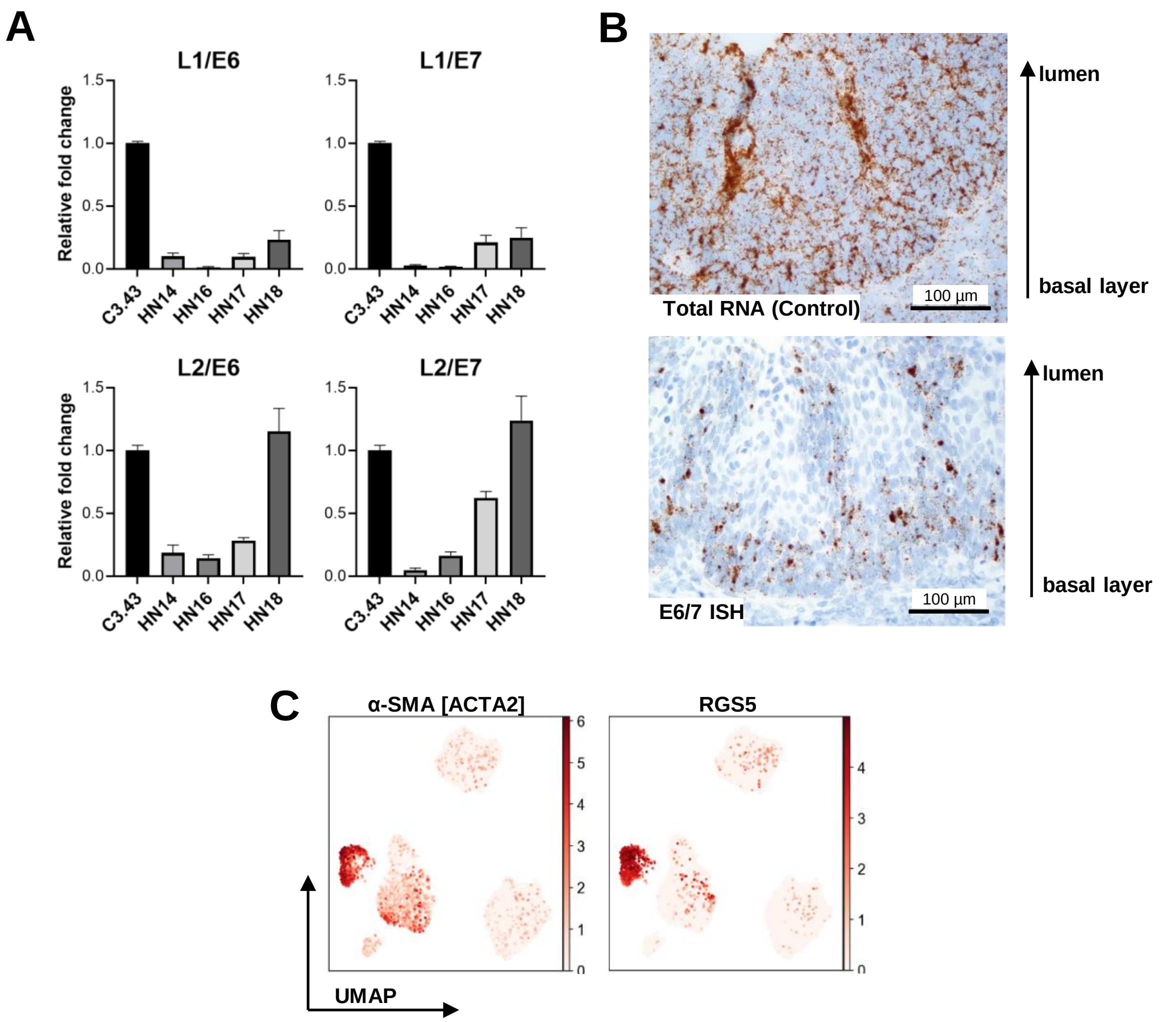

Supplementary Figure 5: Extended data on HPV and pericyte gene expression. (A) RNA expression levels of HPV16 L1 and L2 genes relative to those of E6 or E7 in four HPV+ patients were examined by qPCR. C3.43 control cancer cell line was used as a reference. Tests were done in triplicates. Histograms represent mean and whiskers represent standard error of mean values. (B) Representative *in situ* hybridization images for high-risk HPV types (n=3): upper image depicts 600x magnification of total RNA control showing well-preserved RNA in all cells; lower image represents 600x magnification of E6/E7 mRNA labeling showing a stratified arrangement of expression. (C) UMAP plots showing gene expression of ACTA2 and RGS5, hallmark genes of activated pro-tumor type-2 pericytes. Color bar indicates normalized gene expression.

Supplementary Figure 6

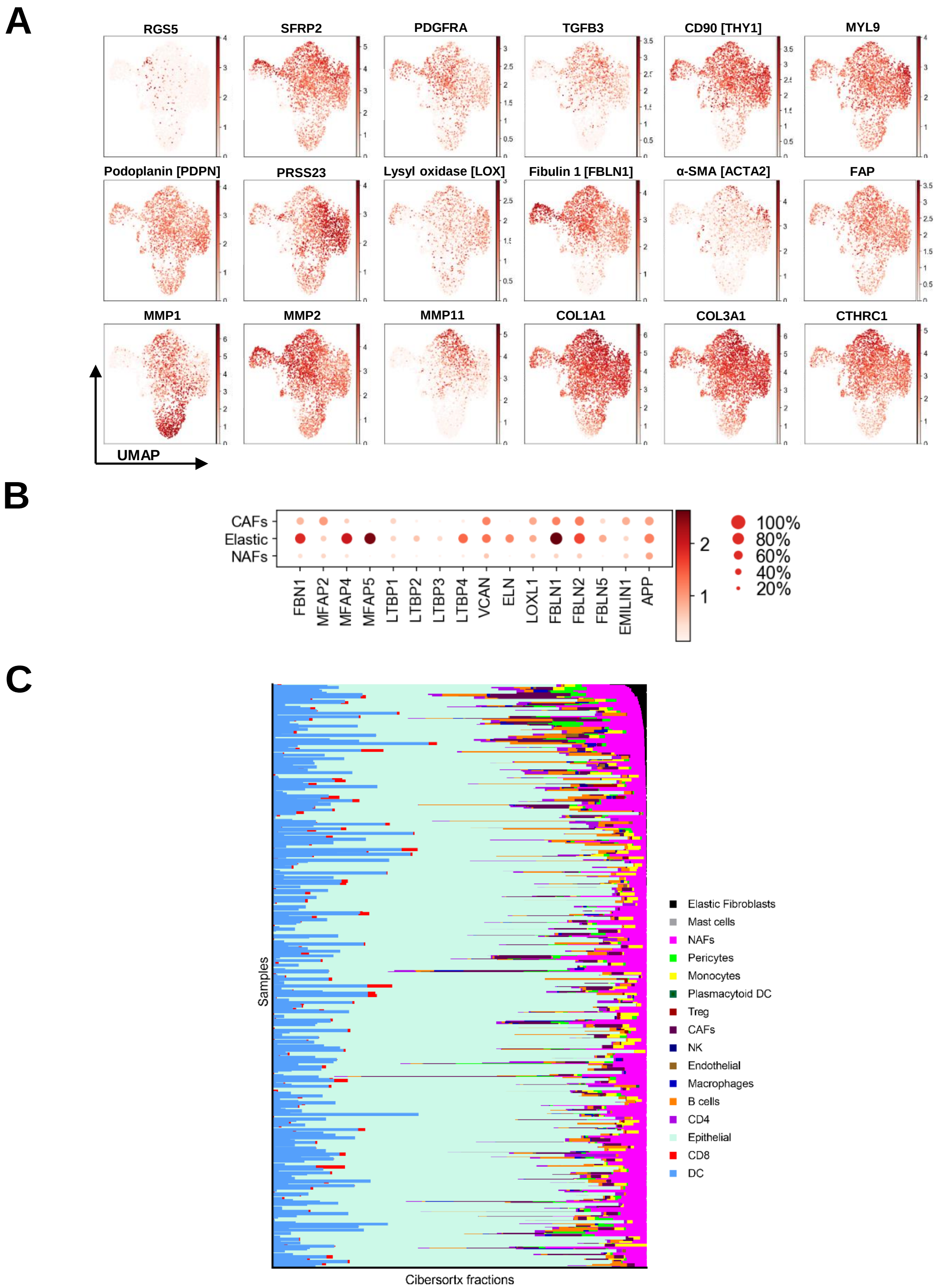

Supplementary Figure 6: Extended data on fibroblast substates. (A) UMAP plots showing genes associated with fibroblast differentiation and activation (n=15 patients). Color bar indicates normalized gene expression (B) Dot-plot depicting the presence of genes associated with elastic differentiation of fibroblasts. (C) CIBERSORTx output showing the predicted fraction of cells of each cell type in the bulk RNASeq HNSCC TCGA cohort.

Supplementary Figure 7

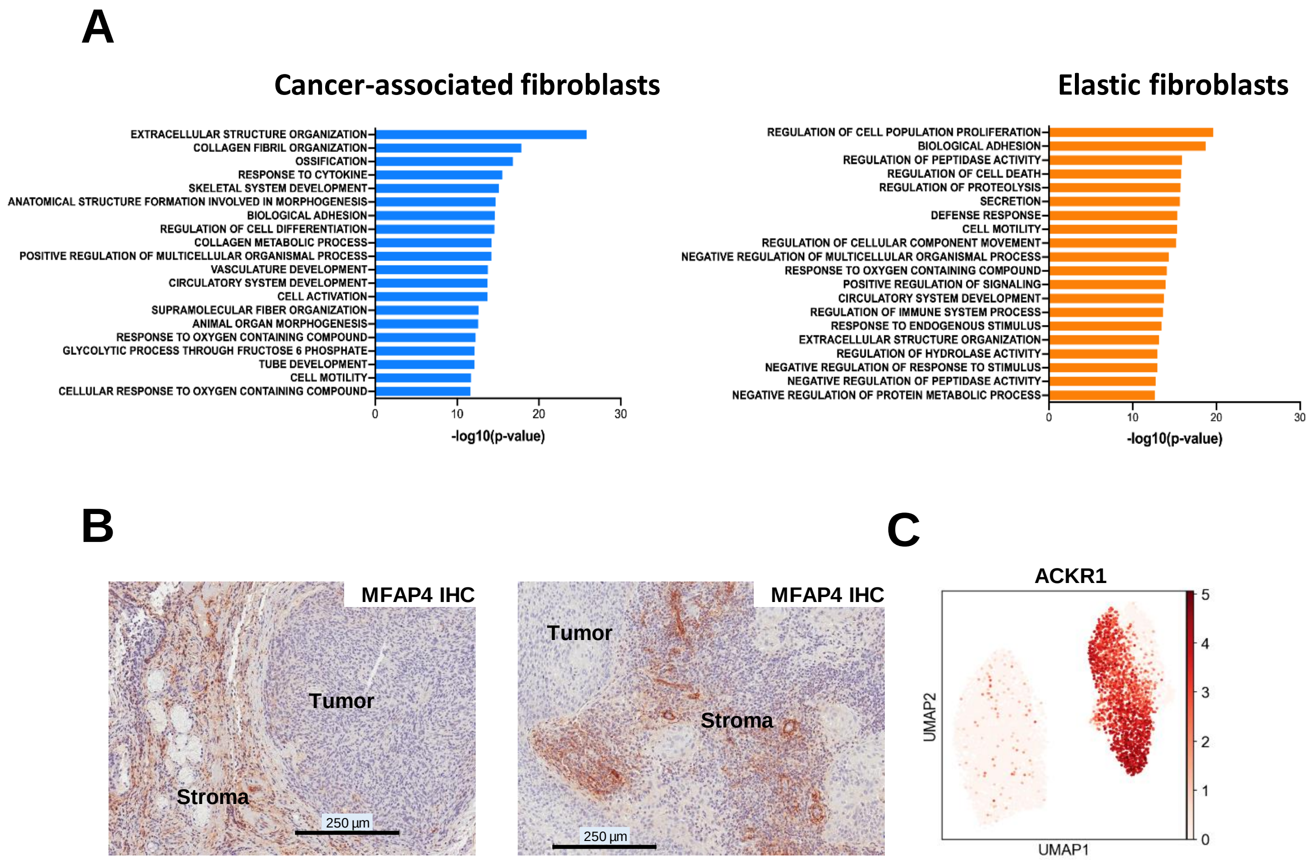

Supplementary Figure 7: Extended data on fibroblast and endothelial cell substates. (A) Gene set expression analysis of CAF or elastic fiber differentiation (GO BP gene set enrichment analysis). (B) Validation of MFAP4 expression by IHC in spindle cells of the tumor stroma in patients (n=6) HN12 (left) and HN17 (right). (C) UMAP plot showing ACKR1 gene expression by endothelial subsets. Color bar indicates normalized gene expression

Supplementary Figure 8

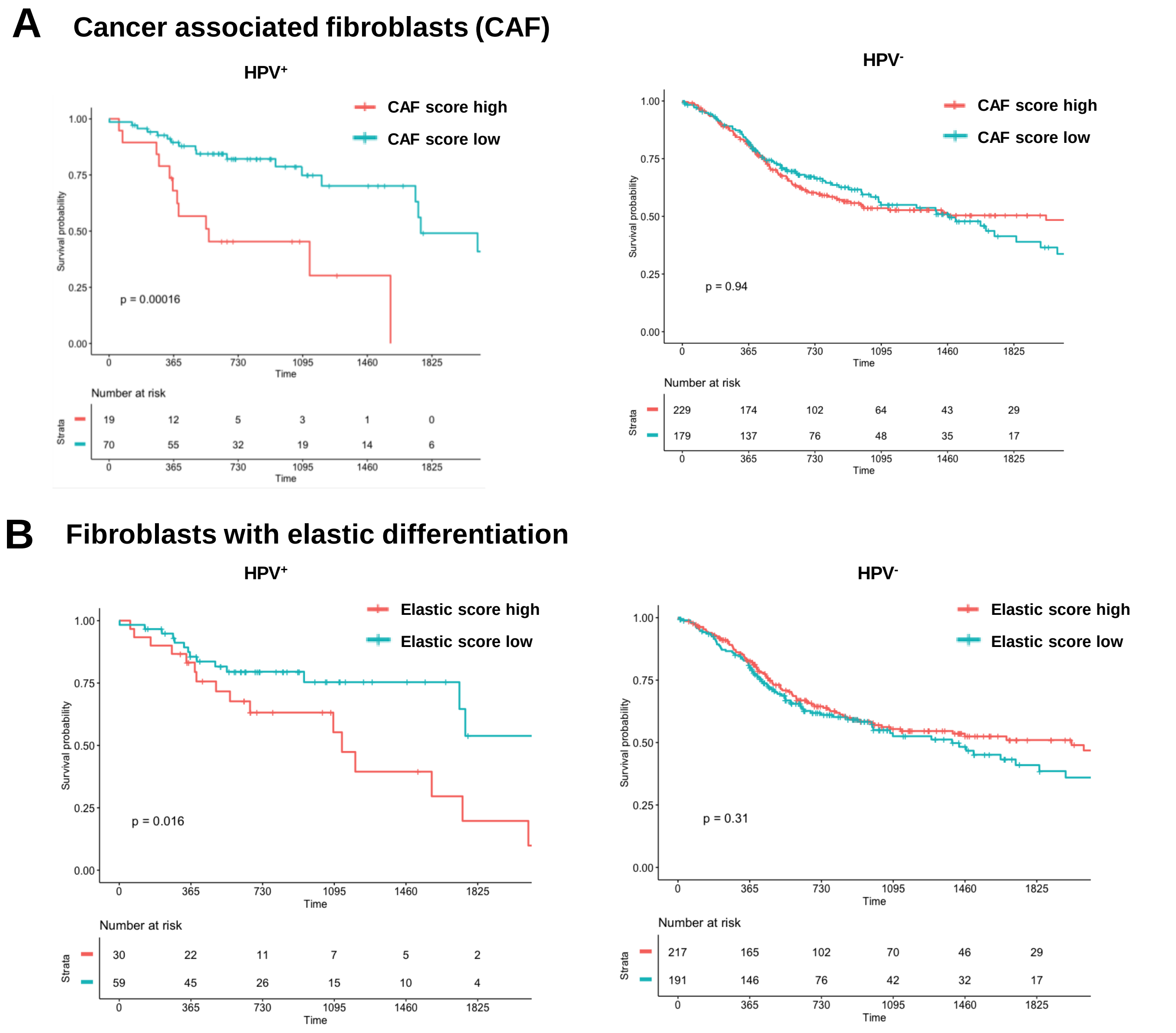

Supplementary Figure 8: Extended data on survival analysis (A) Effect of CAF signature and elastic fibroblast signature (B) on survival in HPV+ and HPV− HNSCC cancers (TCGA). P values calculated using log rank test.

Supplementary Figure 9

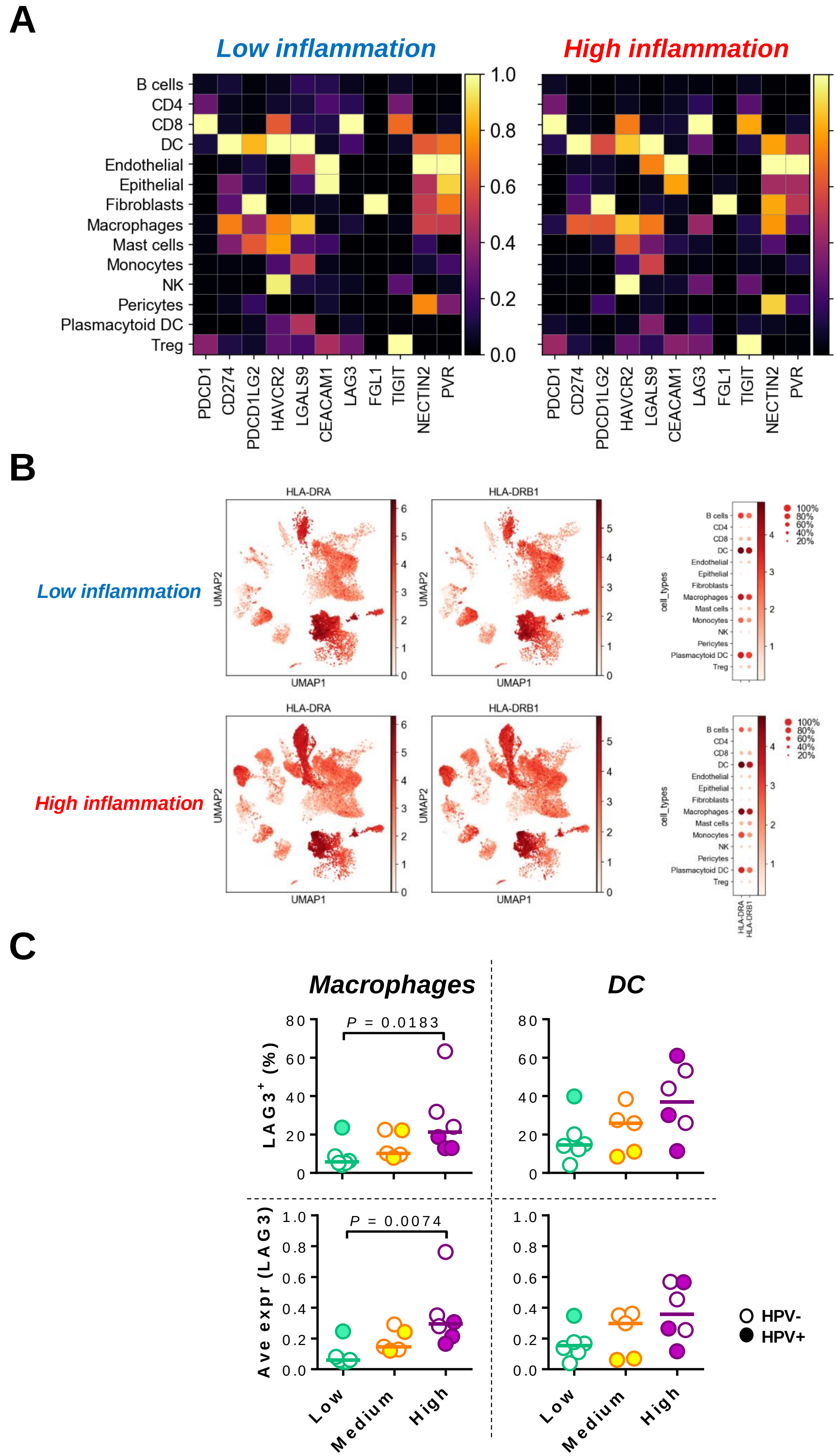

Supplementary Figure 9: Expanded evaluation of ICR and corresponding ICL landscape in tumors with low and high inflammation scores. A) Scaled expression values (denoted by color bar) of genes seen in **Fig. 7A**. B) Relative expression levels for HLA-DRA and HLA-DRB1, prototypical LAG3 ligands, in tumors with low and high inflammation scores are presented. C) Patient-specific frequencies and average expression levels of LAG3 in macrophages and DC according to scRNAseq (n=17 patients, n= 6 Low, n=5 Medium, n=6 High) are shown. Center lines represent median values for each cohort. Data were evaluated using the Kruskal-Wallis and Dunn's multiple comparisons tests.

Supplementary Figure 10

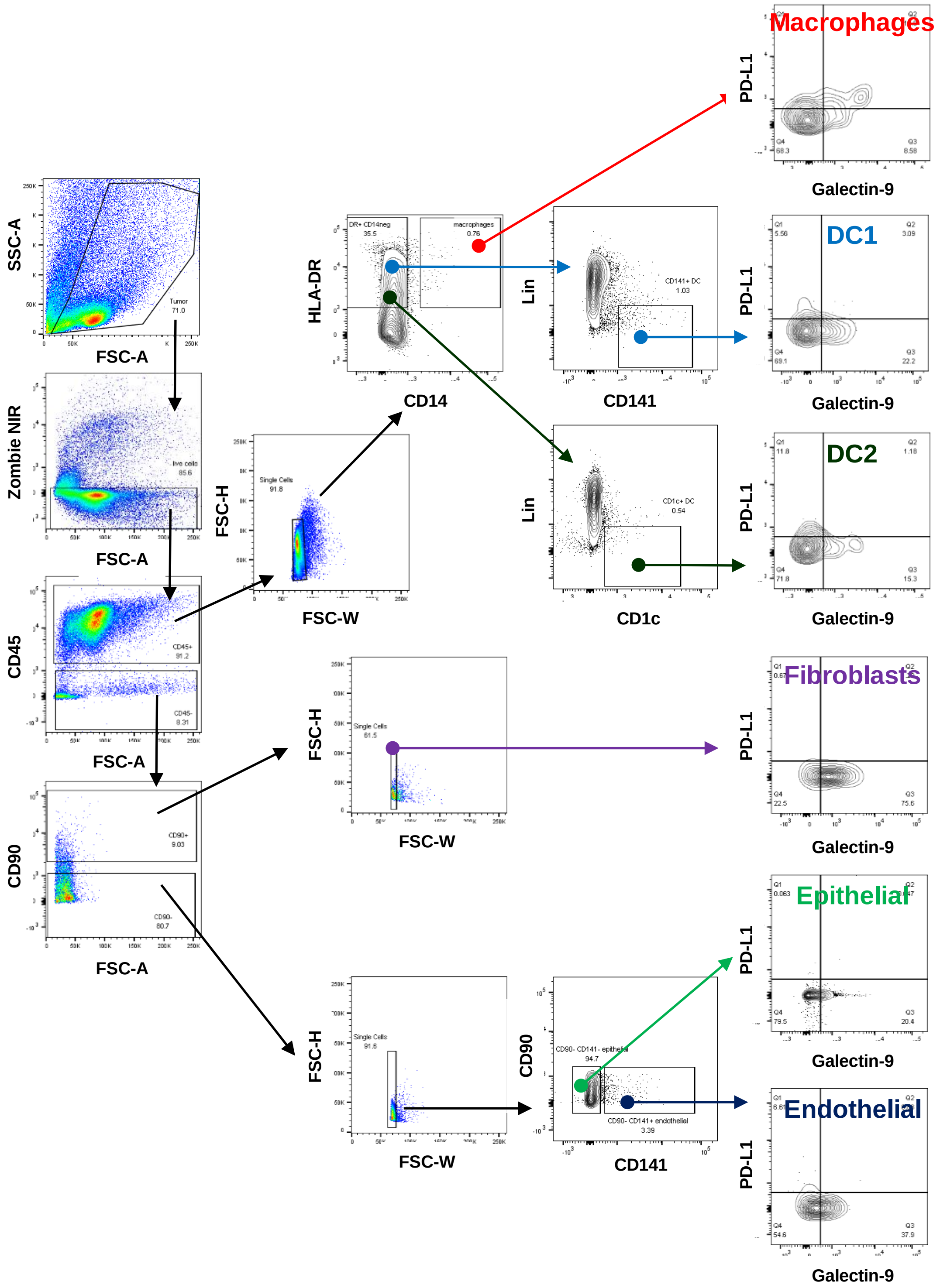

Supplementary Figure 10: Gating and analysis strategy for PD-L1 expression data summarized in Fig. 9A. Staining was performed as described in *Materials and Methods*. Analysis of specific cell populations described in *Results* was performed on Zombie NIR- (viable) CD45+ (leukocytes) and CD45- (non-immune) single cells.

Supplementary Figure 11

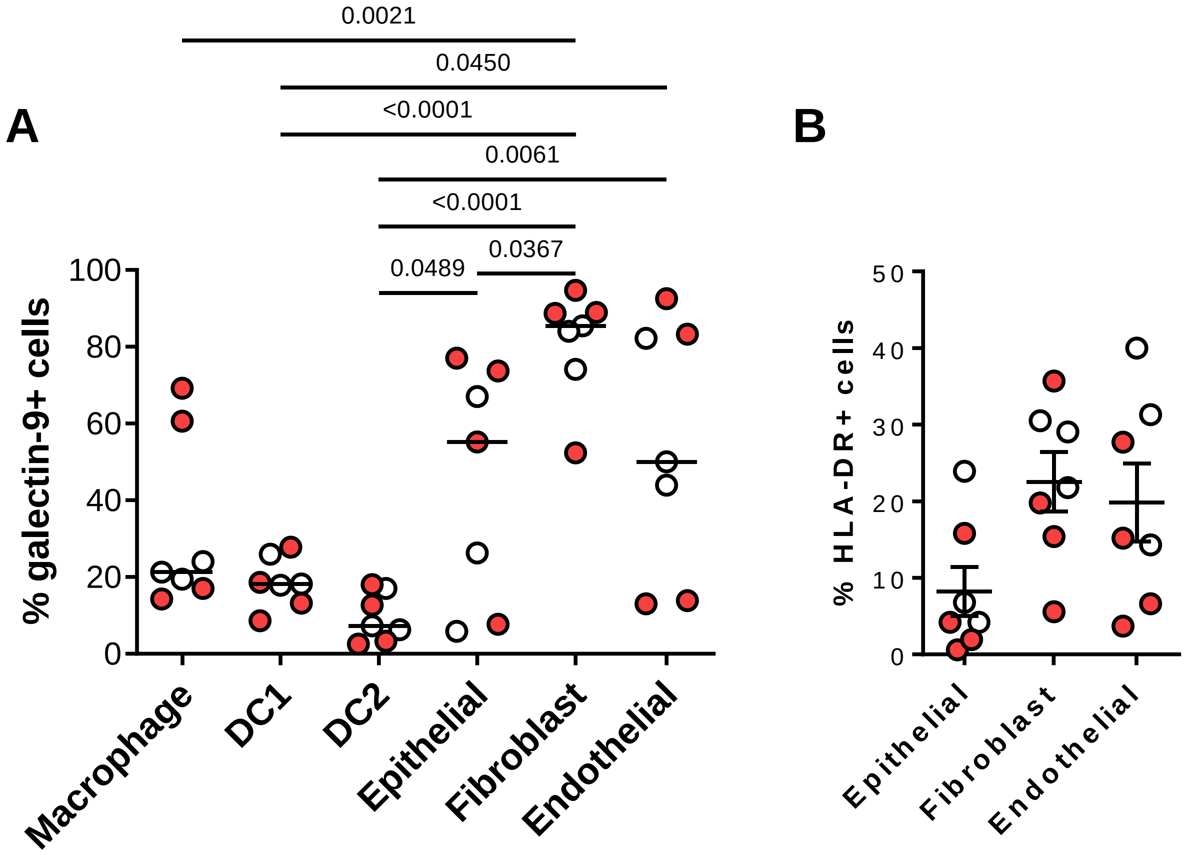

Supplementary Figure 11: Flow cytometric validation of galectin-9 and HLA-DR expression patterns. Expression patterns of A) galectin-9 and B) HLA-DR on myeloid and non-immune cells in the HNSCC TME (n=7) based on flow cytometry (**Suppl. Fig. 9**) are summarized. Center lines represent mean values and whiskers depict standard errors of means. Red circles – HPV^{+} patients. One-way ANOVA test was used for statistical comparisons. *P*-values are depicted.

Supplementary Figure 12

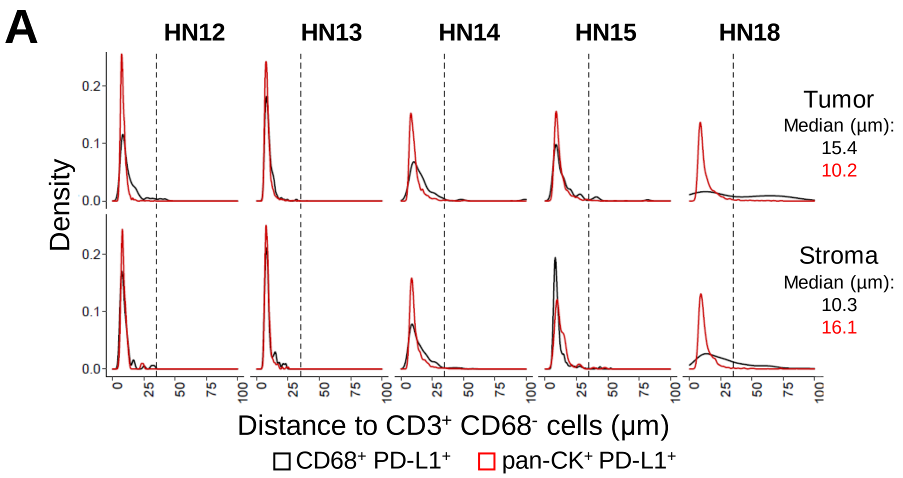

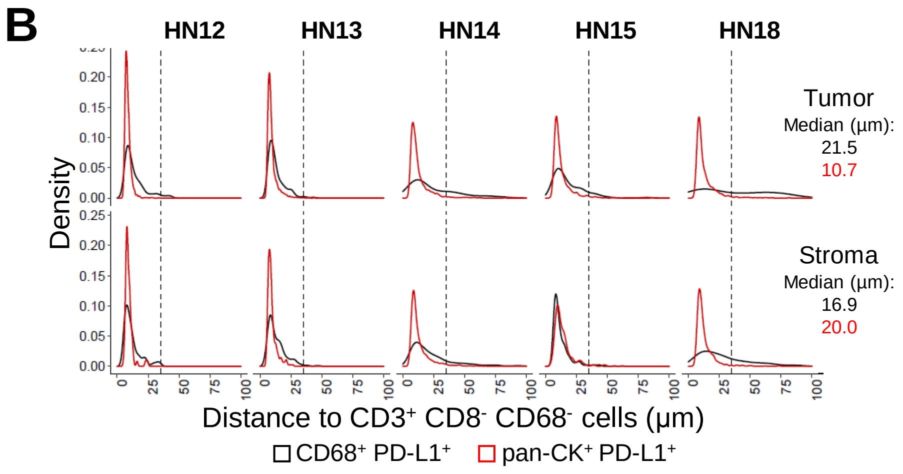

Supplementary Figure 12: Immunofluorescence analyses of T-cell distances to PD-L1^{+} cells within the TME. Distance to (A) total CD3^{+}, as well as (B) CD3^{+} CD8^{-} CD68^{-} T-cells from CD68^{+} PD-L1^{+} macrophages and pan-CK^{+} PD-L1^{+} tumor cells is measured by multispectral microscopy. Data are pooled from all patient-associated ROIs. Calculated median distances across all patients and between evaluated cell types are shown. Dashed lines represent the 35 µm distance used as the cutoff to measure cell-to-cell interactions between evaluated cell types.

Supplementary Table 1. HNSCC patient information: Patient demographics and HPV status of patients included in the cohort for scRNASeq and Flow cytometry validation.

HN #	Gender	Age group	Smoking	Alcohol	Disease site	*T-Stage	*N-Stage	*M-Stage	HPV (p16 IHC)	Inflam. status	Analysis
1	M	70-79	Yes	No	Oral cavity	T4A	N2B	M0	Neg	High	scRNAseq, IHC
2	F	60-69	No	No	Oral cavity	T3	N2a	M0	Neg	Low	scRNAseq
3	M	80-89	No	No	Oral cavity	T4a	N0	M0	Neg	NA	scRNAseq
4	M	50-59	Yes	Yes	Oral cavity	T3	N1	M0	Neg	Low	scRNAseq
5	F	50-59	Yes	Yes	Oral cavity	†T3	†N3b	†M0	Neg	Med	scRNAseq
6	M	30-39	Yes	Yes	Oral cavity	T3	N0	M0	Neg	High	scRNAseq
7	M	60-69	Yes	Yes	Larynx	T3	N0	M0	Neg	Low	scRNAseq
8	F	70-79	Yes	Yes	Oral cavity	†T1	†N0	†M0	Neg	Med	scRNAseq, IHC
9	F	70-79	Yes	Yes	Oral cavity	T3	N2B	M0	Neg	Med	scRNAseq, IHC, Vectra
10	M	50-59	No	Yes	Oral cavity	T3	N0	M0	Neg	High	scRNAseq
11	M	80-89	No	No	Oral cavity	T2	N0	M0	Neg	Low	scRNAseq
12	M	50-59	Yes	Yes	Oropharynx	T2	N1	M0	Pos	Med	scRNAseq, IHC, Vectra
13	M	70-79	No	No	Oropharynx	T2	N0	M0	Pos	High	scRNAseq, IHC, Vectra
14	M	50-59	Yes	Yes	Oropharynx	T1	N1	M0	Pos	High	scRNAseq, IHC, Vectra
15	F	60-69	Yes	NA	Oral cavity	T2	N0	M0	Neg	Low	scRNAseq, IHC, Vectra
16	M	40-49	Yes	Yes	Oropharynx	T2	N1	M0	Pos	Med	scRNAseq, IHC
17	M	50-59	Yes	Yes	Oropharynx	T1	N1	M0	Pos	High	scRNAseq, IHC
18	M	50-59	Yes	Yes	Oropharynx	T2	N2	M0	Pos	Low	scRNAseq, IHC, Vectra
19	F	60-69	Yes	No	Oropharynx	T3	N2B	M0	Neg	NA	Flow
20	M	60-69	NA	No	Oropharynx	T2	N2B	M1	Neg	NA	Flow
21	M	50-59	No	No	Oropharynx	T4	N1	M0	Pos	NA	Flow
22	F	60-69	No	Yes	Oral cavity	T4A	N2C	M0	Neg	NA	Flow
23	M	50-59	Yes	Yes	Oropharynx	T1	N1	M0	Pos	NA	Flow
24	M	50-59	Yes	Yes	Oropharynx	T2	N1	M0	Pos	NA	Flow
25	M	50-59	Yes	Yes	Oropharynx	T2	N1	M0	Pos	NA	Flow

***TNM Eight Edition AJCC guidelines for clinical staging (with exception of patients 5 and 8)**

†Pathologically staged

NA - not available

IHC - immunohistochemistry fibroblast staining

Vectra - multispectral imaging

Supplementary Table 2. MFAP4 scoring.

Case #	**Staining Intensity**	**Percentage (positive cells/all stromal cells)**	**Final Score**	**Note**
HN01	2	1	3	No tumor tissue on this slide. MFAP4 was expressed mainly in the spindle cellular matrix tissues, but not in the normal epithelial cells.
HN08	1	1	2	MFAP4 was expressed mainly in the spindle cellular matrix surrounding tumor tissues and stromal tissues, but not in the tumor cells or normal epithelial cells.
HN09	2	1	3	MFAP4 was expressed mainly in the spindle cellular matrix surrounding tumor tissues and stromal tissues, but not in the tumor cells or normal epithelial cells.
HN12	3	2	5	MFAP4 was expressed mainly in the spindle cellular matrix surrounding tumor tissues and stromal tissues, but not in the tumor cells or normal epithelial cells.
HN16	2	2	4	MFAP4 was expressed mainly in the spindle cellular matrix surrounding tumor tissues and stromal tissues, but not in the tumor cells or normal epithelial cells.
HN17	3	3	6	MFAP4 was expressed mainly in the spindle cellular matrix surrounding tumor tissues and stromal tissues, but not in the tumor cells or normal epithelial cells.

MFAP4 scoring (Staining Intensity: 0, negative; 1, weak staining/trace; 2, moderate staining; 3, strong staining. Percentage: 0, < 0-10%; 1, 11-50%, 2, 51-80%; 3, >80%, Final Score = Intensity + Percentage)

Supplementary Table 3. Detailed list of antibodies and dilutions used for multicolor flow cytometry staining panels.

Antigen	Ab Clone	Fluorochrome	Vendor	Catalog #	Dilution	Panels used
CD45	HI30	PE	BioLegend	304008	1:20	FACS sorting
CD1c	L161	BV510	BioLegend	331534	1:20	Myeloid/non-immune panel
CD14	M5E2	BV711	BioLegend	301838	1:20	Myeloid/non-immune panel
HLA-DR	L243	PerCP-Cy5.5	BioLegend	307630	1:20	Myeloid/non-immune panel
Galectin-9	9M1-3	APC	BioLegend	348908	1:20	Myeloid/non-immune panel
PD-L1	29E.2A3	BV421	BioLegend	329714	1:20	Myeloid/non-immune panel
CD45	HI30	BUV395	BD Bioscience	563792	1:20	Myeloid/non-immune panel
CD90	5E10	PE-Cy7	BD Bioscience	561558	1:20	Myeloid/non-immune panel
CD141	1A4	BB515	BD Bioscience	565084	1:20	Myeloid/non-immune panel
CD3	*UCHT1*	*BUV737*	*BD Bioscience*	*612750*	*1:20*	*Myeloid/non-immune panel*
CD19	*SJ25C1*	*BUV737*	*BD Bioscience*	*612756*	*1:20*	*Myeloid/non-immune panel*
CD56	*NCAM16.2*	*BUV737*	*BD Bioscience*	*612766*	*1:20*	*Myeloid/non-immune panel*

Italicized antigens and antibodies were used to generate the lineage cocktail used for the myeloid/non-immune panel

Supplementary Table 4. Detailed list of antibodies and dilutions used for multispectral immunohistochemistry staining of slides as shown in Figure 8.

Cycle	Antigen retrieval buffer	Antibody -clone (dilution)	Company (Cat#)	Incubation time (min)	Opal (dilution)
1	AR9	**CD3epsilon** - D7A6E (1:200)	Cell Signaling Technology (85061S)	30	520 (1:100)
2	AR9	**CD8**- C8/144B (1:200)	Biocare Medical (ACI3160A)	30	570 (1:100)
3	AR9	**PD-L1**- E1L3N (1:200)	Cell Signaling Technology (13684S)	40	540 (1:100)
4	AR6	**CD68** - D4B9C (1:800)	Cell Signaling Technology (76437S)	30	650 (1:150)
5	AR6	**PanCK**- AE1/AE3 (1:200)	Santa Cruz Biotech (SC81714)	30	690 (1:100)
6	AR6	**DAPI**	Akoya Biosciences (NEL811001KT)	3	None
