# Supplementary material for: Investigating immune and non-immune cell interactions in head and neck tumors by single-cell RNA sequencing
Source: Nat Commun. 2021 Dec 17;12:7338. doi: 10.1038/s41467-021-27619-4 (PMC8683505; doi:10.1038/s41467-021-27619-4)
Supplement: Supplementary file 2 — Description of Additional Supplementary Files [file 41467_2021_27619_MOESM2_ESM.pdf]

Title: Supplementary Data 1:

Description: Top 100 differentially expressed genes when comparing each fibroblast type with the others. CAF = cancer associated fibroblasts, NAF = normal activated fibroblasts, grey background signifies these genes also being shown in Suppl. Fig. S6B
