# Supplementary material for: Investigating immune and non-immune cell interactions in head and neck tumors by single-cell RNA sequencing
Source: Nat Commun. 2021 Dec 17;12:7338. doi: 10.1038/s41467-021-27619-4 (PMC8683505; doi:10.1038/s41467-021-27619-4)
Supplement: Supplementary file 4 — Reporting Summary [file 41467_2021_27619_MOESM4_ESM.pdf]

nature research

Corresponding author(s): Dr. Robert L. Ferris

Last updated by author(s): Nov 7, 2021

Reporting Summary

Nature Research wishes to improve the reproducibility of the work that we publish. This form provides structure for consistency and transparency in reporting. For further information on Nature Research policies, see Authors & Referees and the Editorial Policy Checklist .

Statistics

For all statistical analyses, confirm that the following items are present in the figure legend, table legend, main text, or Methods section.

n/a	Confirmed	
☐	☒	The exact sample size (*n*) for each experimental group/condition, given as a discrete number and unit of measurement
☐	☒	A statement on whether measurements were taken from distinct samples or whether the same sample was measured repeatedly
☐	☒	The statistical test(s) used AND whether they are one- or two-sided<br>*Only common tests should be described solely by name; describe more complex techniques in the Methods section.*
☒	☐	A description of all covariates tested
☒	☐	A description of any assumptions or corrections, such as tests of normality and adjustment for multiple comparisons
☐	☒	A full description of the statistical parameters including central tendency (e.g. means) or other basic estimates (e.g. regression coefficient) AND variation (e.g. standard deviation) or associated estimates of uncertainty (e.g. confidence intervals)
☐	☒	For null hypothesis testing, the test statistic (e.g. *F*, *t*, *r*) with confidence intervals, effect sizes, degrees of freedom and *P* value noted<br>*Give P values as exact values whenever suitable.*
☒	☐	For Bayesian analysis, information on the choice of priors and Markov chain Monte Carlo settings
☒	☐	For hierarchical and complex designs, identification of the appropriate level for tests and full reporting of outcomes
☒	☐	Estimates of effect sizes (e.g. Cohen's *d*, Pearson's *r*), indicating how they were calculated

Our web collection on statistics for biologists contains articles on many of the points above.

Software and code

Policy information about availability of computer code

Data collection	Data were acquired using LSR Fortessa II cytometer with BD FACSDiva (v.9.0) software (BD Biosciences)
Data analysis	No custom code was used for this analysis. Open source software used (details of usage in the methods section) are Cellranger (v3.0.0), Scanpy (v1.4.5.post2), singleseqgset R package(v0.1.0.9000), infercnv (v1.2.1), FlowJo (v10.6.1), cellphonedb(v2.1.4),inForm(v2.4.6) by Akoya Biosciences, Phenochart(v1.0),QuPath(v0.2.3), survival R package(v.3.2-11).

For manuscripts utilizing custom algorithms or software that are central to the research but not yet described in published literature, software must be made available to editors/reviewers. We strongly encourage code deposition in a community repository (e.g. GitHub). See the Nature Research guidelines for submitting code & software for further information.

Data

Policy information about availability of data

All manuscripts must include a data availability statement. This statement should provide the following information, where applicable:

- Accession codes, unique identifiers, or web links for publicly available datasets
- A list of figures that have associated raw data
- A description of any restrictions on data availability

Raw data are available on NCBI Sequence Read Archive Accession ID SRP301444 (https://www.ncbi.nlm.nih.gov/sra?term=SRP301444). Processed gene barcode are available on the Gene Expression Omnibus database: Accession ID GSE164690 (https://www.ncbi.nlm.nih.gov/geo/query/acc.cgi?acc=GSE164690). The bulk RNAseq and clinical HNSCC data utilized for survival analysis and deconvolution using Cibersort from TCGA is available through the Broad Genome Data Analysis Center Firehouse (https://gdac.broadinstitute.org/). Gene signatures from the MSigDB can be found on their website (http://www.gsea-msigdb.org/gsea/msigdb)

Field-specific reporting

Please select the one below that is the best fit for your research. If you are not sure, read the appropriate sections before making your selection.

- [x] Life sciences
- [ ] Behavioural & social sciences
- [ ] Ecological, evolutionary & environmental sciences

For a reference copy of the document with all sections, see nature.com/documents/nr-reporting-summary-flat.pdf

Life sciences study design

All studies must disclose on these points even when the disclosure is negative.

Sample size	We utilized 2 in-house generated and 1 publicly available HNSCC TCGA cohorts for this study. For the scRNASeq cohort, we retrospectively confirmed that our dataset has an adequate number of PBL and CD45+/- cells sequenced using the SCOPIT algorithm (Davis, A., et al. BMC Bioinformatics. 2019; 20(1): 566.). Mast cells were the rarest cell type identified in both the PBL and tumor cells in our cohort. Using the frequency of mast cells, the number of cells to be sequenced for a 0.95 probability of success was predicted to be 4575 for PBL and 69981 for CD45+ cells and our dataset meets both these thresholds (36,390 cells from PBL and 71,102 CD45+ cells). The flow cytometry cohort used to validate findings from the scRNASeq cohort was chosen based on sample availability.
Data exclusions	HN02-HN04 didn't have enough live CD45n cells in the viability check prior to loading the 10x Controller. Thus they do not contribute to any analysis related to the tumor&stroma. HN03 had no viable tumor cells (only necrosis and stroma) after its use for fresh digestion and scRNAseq, so it was excluded from analysis of inflammation status.
Replication	Due to limited sample availability, scRNASeq experiments were performed once per patient.
Randomization	Not relevant to our non-interventional study. Samples were obtained consecutively as the specimen became available during surgical treatment.
Blinding	This is an exploratory study of the HNSCC tumor microenvironment. Since the samples were treatment naive, blinding was not applicable to this study.

Reporting for specific materials, systems and methods

We require information from authors about some types of materials, experimental systems and methods used in many studies. Here, indicate whether each material, system or method listed is relevant to your study. If you are not sure if a list item applies to your research, read the appropriate section before selecting a response.

Materials & experimental systems

n/a	Involved in the study	
[ ]	[x]	Antibodies
[x]	[ ]	Eukaryotic cell lines
[x]	[ ]	Palaeontology
[x]	[ ]	Animals and other organisms
[ ]	[x]	Human research participants
[x]	[ ]	Clinical data

Methods

n/a	Involved in the study	
[x]	[ ]	ChIP-seq
[ ]	[x]	Flow cytometry
[x]	[ ]	MRI-based neuroimaging

Antibodies

Antibodies used

Antibodies used in IHC:
MFAP4 Polyclonal Rabbit IgG Novus NBP2-30439

Antibodes used for Flow Cytometry:
CD45 HI30 PE BioLegend 304008
CD1c L161 BV510 BioLegend 331534
CD14 M5E2 BV711 BioLegend 301838
HLA-DR L243 PerCP-Cy5.5 BioLegend 307630
Galectin-9 9M1-3 APC BioLegend 348908
PD-L1 29E.2A3 BV421 BioLegend 329714
CD45 HI30 BUV395 BD Bioscience 563792
CD90 5E10 PE-Cy7 BD Bioscience 561558
CD141 1A4 BB515 BD Bioscience 565084
CD3 UCHT1 BUV737 BD Bioscience 612750
CD19 SJ25C1 BUV737 BD Bioscience 612756

CD56 NCAM16.2 BUV737 BD Bioscience 612766

Antibodies used in multispectral imaging:
CD3epsilon - D7A6E Cell Signaling Technology (85061S)
CD8- C8/144B Biocare Medical (ACI3160A)
PD-L1- E1L3N Cell Signaling Technology (13684S)
CD68 - D4B9C (1:800) Cell Signaling Technology (76437S)
PanCK- AE1/AE3 (1:200) Santa Cruz Biotech (SC81714)
DAPI Akoya Biosciences (NEL811001KT)

Validation

All antibodies used in this study are commercially available and validated against human antigens and previous publications. Antibodies were validated by staining single cell suspensions from peripheral blood and/or HNSCC tumors.

Human research participants

Policy information about studies involving human research participants

Population characteristics

Information can be found in Supplemental Table 1

Recruitment

After informed consent, fresh peripheral blood and tumor biopsies were obtained from treatment-naive HNSCC patients. These patients were recruited consecutively. Neither the patient, the surgeon or the tissue bank staff were aware of the details of the experiment or research questions, making a selection bias unlikely.

Ethics oversight

University of Pittsburgh Medical Center Hillman Cancer Center IRB 99-069

Note that full information on the approval of the study protocol must also be provided in the manuscript.

Flow Cytometry

Plots

Confirm that:

- [x] The axis labels state the marker and fluorochrome used (e.g. CD4-FITC).
- [x] The axis scales are clearly visible. Include numbers along axes only for bottom left plot of group (a 'group' is an analysis of identical markers).
- [x] All plots are contour plots with outliers or pseudocolor plots.
- [x] A numerical value for number of cells or percentage (with statistics) is provided.

Methodology

Sample preparation

After informed consent, tumor biopsies were obtained from treatment-naive HNSCC patients. After physical dissociation, tumors underwent a 30 min enzymatic digestion in a dissociation cocktail [1X HBSS supplemented with 50 IU/ml collagenase I, 25 IU/ml collagenase II, 50 IU/ml collagenase IV 0.025 mg/ml DNase I (STEMCELL Technologies; Vancouver, Canada) and 3 mM calcium chloride (Sigma-Aldrich; St. Louis, MO)] at 37C° and cell extraction. Subsequently samples were washed and stained in FACS buffer.

Instrument

BD LSR Fortessa II

Software

BD FACSDiva for sample collection
FlowJo v10.6.1 for data analysis

Cell population abundance

Immune and non-immune cells were sorted using a highly conservative gating strategy. Post-sort and post scRNAseq purity of cells was validated bioinformatically using PTPRC expression

Gating strategy

Cells were first gated on FSC-A and SSC-A to select total cell population. Next the cells were gated on FSC-A and Zombie NIR viability dye to exclude dead cells. Single cells were then gated via CD45 expression to separate immune cells from the non-immune cell population and FSC-W and FSC-H to exclude doublets. For immune cell analysis, macrophages were gated as HLA-DR + CD14+, DC1 cells as HLA-DR+CD14- Lin(CD3/CDC19/CD56)- CD141+, and DC2 as HLA-DR+CD14- Lin(CD3/CDC19/CD56)- CD1c+. For non-immune cell analysis, fibroblasts were characterized as CD45-CD90+, epithelial cells as CD45-CD90- and endothelial cells as CD45-CD90-CD141+. Positive expression of PD-L1 on different cell populations was analyzed based on isotype control.

- [x] Tick this box to confirm that a figure exemplifying the gating strategy is provided in the Supplementary Information.
